# A Helpline Telephone Service for Tobacco Related Issues: The Italian Experience

**DOI:** 10.3390/ijerph6030900

**Published:** 2009-02-26

**Authors:** Enrica Pizzi, Alessandra Di Pucchio, Luisa Mastrobattista, Renata Solimini, Roberta Pacifici, Simona Pichini

**Affiliations:** Therapeutic Research and Medicines Evaluation Department, Italian Epidemiological Observatory on Tobacco, Alcohol and Drugs of abuse, Istituto Superiore di Sanità, Viale Regina Elena 299, 00161 Rome, Italy; E-Mails: alessandra.dipucchio@iss.it (A.D.P.); luisa.mastrobattista@iss.it (L. M.); renata.solimini@iss.it (R.S.); roberta.pacifici@iss.it (R.P.); simona.pichini@iss.it (S. P.)

**Keywords:** Italian Antismoking Helpline, counselling, tobacco smoking

## Abstract

Antismoking helplines have become an integral part of tobacco control efforts in many countries, including Italy. The demonstrated efficacy and the convenience of telephone based counselling have led to the fast adoption of antismoking helplines. However, information on how these helplines operate in actual practice is not often readily available. This paper provides an overview of the Italian Antismoking Helpline, an increasingly popular telephone service for tobacco problems operating in Italy since 2000. As many states, regions and nations are contemplating various telephone programs as part of large scale anti-tobacco campaigns, this paper briefly discusses the reasons the helpline is well suited to lead the cessation component of a comprehensive tobacco control program, how it operates and how it can be used in conjunction with other tobacco control activities. The Italian Antismoking Helpline provides Italians with free services that include counselling, cessation related information, self help quit kits and current legislation information. The helpline is promoted statewide by media campaigns, health care providers, local tobacco control programs and public school system. The Helpline is centrally operated through the Istituto Superiore di Sanità and it has served over 17.000 tobacco users and others.

## 1. Introduction

Today the scientific community agrees that tobacco smoking is the main cause of morbidity and avoidable mortality [1]. Smoking prevention and the fight against tobacco consumption are priority objectives of the health policies in the international community and in our country [2].

The main strategies regarding tobacco control suggested by the U.S. Surgeon General in 2000 were: the reduction of exposure to environmental tobacco smoke; the reduction of tobacco use initiation and the increase of tobacco use cessation [3].

Other general recommendations included educational strategies, management of nicotine addiction, regulatory efforts (advertising and promotion, product regulation, clean indoor air regulation, minors’ access to tobacco, litigation approaches), economic approaches, comprehensive programs, global efforts, and elimination of health disparities.

In this context, Italy has started to implement in recent years the international strategies for tobacco control, with the Italian Epidemiological Observatory on Tobacco, Alcohol and Drugs of abuse of the Istituto Superiore di Sanità (OssFAD, ISS), playing a key role in the achievement of the above-mentioned objectives [4].

The OssFAD, set up at the Istituto Superiore di Sanità since 2000 at the request of the former Ministry of Health, is the official organ for information concerning nicotine addiction, alcoholism and drug addiction. It provides health and legal information, scientific data, reviews, updates and meetings on related matters.

An antismoking helpline was set up by OssFAD to help smokers who want to quit the habit, to support the people in their fight against second-hand smoke, and to facilitate health promotion activities. In fact a lot of national and international experiences show that helplines are valid tools in supportof public health interventions [5].

In this report we describe the main activities of the Italian Antismoking Helpline that has now been in operation for over eight years and has served more than 17.000 people in Italy. As many decision makers are weighing the value of tobacco helplines and others are in the process of implementing one, this paper aims to provide information that will be useful for policy makers as well as for practitioners and researchers. The potential of such a helpline for future tobacco control practice and research is also discussed.

## 2. Results and Discussion

### 2.1. The Smoking Problem

The prevalence of active smokers in Italy is still very high (26.4% and 17.9%, respectively, for men and for women over 15 years of age) [6]. About 80,000 annual deaths are attributable to tobacco smoking in Italy (of which approximately 48% is due to cancer, 25% to cardiovascular disease, and 17% to respiratory disease) [7].

More than 25% of the deaths attributable to smoking occur in people between the ages of 35 and 65. Smoking is detrimental at any age, but the correlated risks of developing pathologies (cardiovascular, oncological, and respiratory) are closely correlated to the age the habit started. For example, a person who starts smoking at age 15 is three-times more likely to develop a cancer compared to an individual who starts smoking at age 20 [8,9].

Exposure to passive smoke causes an increased risk for respiratory disease (particularly in children) [10], for myocardial infarction [11] and for lung cancer [12]. Mothers’ smoking during pregnancy is the cause of many pathologies, with serious consequences on the neurobehavioral development of the infant; among the most prominent consequences to note is the significant reduction in birth weight [13] and an excess risk of sudden infant death (SIDS) [14]. Despite such epidemiological data, the negative effects of smoking in Italy are still underestimated by both the general population and health professionals.

### 2.2. Telephone Counsellingand t he Italian Antismoking Helpline’s Experience

Telephone-based tobacco cessation services, commonly known as quitlines or helplines, have many advantages that have made them a top cessation and health promotion strategies for several countries (e.g., USA, Australia, England, etc.). These advantages have led the Interagency Committee on Smoking and Health, Cessation Subcommittee, to recommend the establishment of a national network of state-managed quitlines to provide universal coverage for tobacco cessation [15].

Their effectiveness with smokers who use them is well established [16–18] and in many nations with comprehensive tobacco control programs, heplines play an integral role in media-based efforts to increase quittingattempts and to disseminate tobacco -related issues to the general population [19].

Consequently, recognising the potential of a centralised telephone service, several countries in the world have established nationwide tobacco helplines such as: Australian Quitline (*from 1989*), Scotland Antismoking Telephone Helpline, California Smokers’ Helpline (*from 1992*), Massachussetts Quitline, Oregon Quitline, Arizona Smokers’Helpline, Nevada Tobacco User's Helpline, Canadian Toll-free Quitlines, England Telephone Helpline (*from 1994*) [20]. In Italy, the Antismoking Helpline was set up in 2000, within the framework of the Italian Epidemiological Observatory on Tobacco, Alcohol and Drugs of abuse located at the Istituto Superiore di Sanità. All of these helplines have their own specific characteristics that depend on the geographical place, on the professionals involved and on the specific objectives of the service.

Indeed, the majority of the above-mentioned helplines provide services through proactive counselling and in this case the counsellors use outbound calls. The outbound service, which often entails multiple follow-up sessions, is typically scheduled by agreement with the smoker. In this concern, the efficacy of such proactive interventions has been established by randomized, controlled trials [18]. Differently, the Italian Antismoking Helpline provides services through reactive counselling, answering to callers’ immediate requests for assistance but does not provide outbound counselling calls.

In general, the helplines have many advantages, such as their accessibility. A telephone operation eliminates many of the barriers of traditional cessation classes, such as having to wait for classes to form or needing to arrange for transportation. For example as evidence of the greater accessibility of helplines, surveys have indicated that smokers are several times more likely to use such a service than they are to use a face-to-face program [21,22].

Due to their quasi-anonymous nature, telephone services may also appeal to those who are reluctant to seek help provided in a group setting, helping them overcome what can be a significant psychological barrier [22].

Moreover, populations that are underrepresented in traditional cessation services, such as smokers of ethnic minority backgrounds, actively seek help from quitlines. Helplines are particularly helpful for people with limited mobility and those who live in rural or remote areas [23].

Another advantage of antismoking helplines is that the centralized nature of their operations creates opportunities for efficiency in executing the cessation component of a state’s tobacco control program. A single large-scale promotional campaign for a statewide quitline is more feasible than numerous smaller campaigns for a wide range of local programs. A centralized quitline can also serve as an information clearinghouse and provide direct referrals to local programs for callers who want to use them [19].

The Italian Antismoking Helpline, offered free of charge to all Italians, includes individual counselling, self help materials, information related to tobacco cessation, referral to local services and information about current legislation. It is important to underline that the Italian Helpline has been set up not only to address smokers toward cessation services (SCS), but also to provide useful counselling and information to various groups of targets (Table 1).

The service operates five days a week (Monday to Friday) between 10 a.m. and 4 p.m. (working hours of the Public Institution which hosts the helpline), qualified counsellors provide service [24]. Voice mails are recorded during non-operation hours and public holidays. Overall, the Helpline received over 17,000 calls from May 2000 to December 2008. The Table 2 shows the socio-demographic profile of callers to the helpline and their main questions.

When a call comes into the Helpline, a staff member conducts a brief intake interview, gathering information about the caller (gender, age, geographic range, areas of interest, etc.); all data are then collected by the counsellors, in properly dedicated database. The main areas of interest are defined through the analysis of the main topics coming out during the phone calls.

Many callers are smokers or other tobacco users (61.4%) who are contemplating quitting; in this case the counsellor asks about tobacco use, previous quitting attempts, attitudes about quitting, and demographics. Those who are ready to quit receive information about smoking cessation services (SCS) set up within the Italian National Health Service (Servizio Sanitario Nazionale - SSN) and if they want self help materials, the staff will send it to their home address.

Some callers (9.8%) do not use tobacco themselves but are simply requesting information for a family member or a friend; the counsellor will support such callers in their effort to help the family member or friend. Other callers (7.2%) are people that want advice with respect to exposure to secondhand smoke. In these cases the Helpline Staff gives information on legislation to protect themselves and helps people to put in practice actions to eliminate the passive smoking exposure. Other callers (17.9%) are health care professionals, social workers, public and private institutions who request scientific material or cooperation to carry out studies and to promote health promotion campaigns.

In all these cases the methodology used by Helpline Staff is counselling and orienting the individual in recognizing personal, familiar and environmental resources.

The mean age of the people who contacted the Antismoking Helpline is 42 years. The most frequently represented age groups were 30–39 years (24.3%), 40–49 years (25.9%) and 50–59 years (23.1). The callers were interested in: psychological support, health care information, scientific and legislative information.

With respect to smokers, it has to be acknowledged that people younger than 25 years rarely (less than 5%) call the helpline, although it is know that 24% of smokers are younger than 25 years of age. Furthermore, even if in Italy 57.6% smokers are men and 42.4% are women, these latter are more prone to call the helpline than male smokers (56.3% women *vs* 43.2% men). Finally, the geographic range of callers fairly matches the regional distribution of active smokers.

Overall, about 80% of callers reported that they had heard about the helpline from media sources and about 11% said that their health care providers referred them. About 8% reported that they had heard about the Helpline from family or friends, who may have used the Helpline themselves or simply seen the media spots and passed the information along.

### 2.3. The Italian Antismoking Helpline and Smoking Cessation Services

An important Italian Antismoking Helpline activity is the assessment of the Network of the Italian Cessation Services (SCS). Every year the Helpline Staff carries out an investigation aimed at providing information about structural and organizational characteristics of SCS set up within the Italian National Health Service. To obtain structural and organizational characteristics of smoking cessation services and monitor their activities, telephone interviews are held involving principal coordinators of each Italian SCS and the data is collected using a standard monitoring interview form, entered in a dedicated database and analyzed by SPSS 15.0 software [25].

The last update was carried out in April 2008, a total of 267 SCS were counted in a census: 149 (55.8%) belonging to the Local Care Departments, 109 (40.8%) to hospital’s organizations and 9 (3.4%) to both of them. These services provide different tobacco-use cessation programs. To assist the smokers, 94% of them suggest pharmacotherapies, 83.9% individual counselling and 62.5% group therapy. In addiction, 20% of them provide other treatments such as acupuncture, relaxation therapy and hypnotherapy. However in 97% of SCS, tobacco use cessation programs consist of a combination of the above mentioned therapeutic treatments.

A variety of health care professionals operate in the SCS, 96.8% of services is led by at least one physician who might operate alone (6.4%) or in a team with either clinical psychologists (59.9%) or other health care professionals (30.5%). To access to the tobacco-use cessation programs, 75% of the SCS require some patient contribution (e.g., ticket, association’s fee), 15% are cost-free and 10% a combination of both.

The national-based survey, a uniform method for gathering homogeneous and comparable data is the platform for the development of integrated, coordinated and effective tobacco cessation strategies.

The update allows the OssFAD to prepare a SCS Guide that represents a useful system to collect information that facilitates access to the services, by making the health services action on user demand easier and faster[ 26].

Moreover, the Helpline Staff carries out important research in cooperation with SCS and other Health Services, such as a prospective longitudinal multicentre study involving 41 smoking cessation services in 16 Italian regions to describe the characteristics and effectiveness of various smoking cessation programs offered by SCS.

The study population of 1,226 patients (54.2% males and 45.4% females), mean age 47 years entered smoking cessation programs between April 2003 and June 2004. Patients had a middle/high level of education (24.8% middle school, 46.9%, high school, 18.4% graduated) and a long history of active smoking (29.4 years); the majority were highly dependent on nicotine (23.5 cigarettes/day) and 61.1% of the participants reported previous attempts to quit smoking.

Results showed that at three months follow up, of the 84% participants still in contact with the SCS 45% declared to have quit smoking. At six months follow up, of the 66% participants still in contact with the SCS, 39% declared to have quit smoking and finally at 12 months the proportion of abstinents was still around 40% within contacted participants. Enrolling people in any type of therapeutic program, in particular nicotine replacement therapy combined with group therapy, increased the probability of successfully quitting smoking; moreover, patients that begin a smoking cessation program should be encouraged to complete the therapy. In general, participants who lost contact with the Center resulted more prone to smoking relapse [27].

These studies are helpful for monitoring activity on the qualities and efficacy of therapies provided by different SCSs. The constant monitoring of characteristics and activities is fundamental for the Helpline Staff to increase the access to local services and support efficiently the people who contact the Italian Helpline.

Moreover to support SCS’ activities the Helpline Staff sends them OssFAD materials and scientific disseminations on related-tobacco issues (self help material for smokers, manuals, scientific articles, posters). Indeed, this ongoing relationship between Antismoking Italian Helpline and SCS enables networking between the different structures.

### 2.4. The Antismoking Italian Helpline and OssFAD Material

The OssFAD is continuously publishing information material and self-help publications concerning tobacco smoking and smoking cessation. Some examples of publications are: the “Guidelines to quit smoking habit” aimed for family physicians who wish provide counselling to quit smoking for all the people coming for a visit; “Guidelines to quit smoking habit” for personal use (to be self administered), Risk Charts for chronic obstructive pulmonary disease (COPD) and lung cancer, learning material such as CD, videos, DVD and books for primary and secondary schools, posters and pamphlets.

The Italian Antismoking Helpline has a key role in promoting and providing the above-mentioned kinds of materials. The telephone number (800-554088) of the service is reported with the aim to inform both on its use and on tobacco-related issues.

Particularly in recent years the Helpline Staff has given an important support for the tobacco use prevention projects promoted by OssFAD and addressed to school settings. These projects are a key element in the overall tobacco control program; in fact tobacco use is a crucial problem that typically starts during adolescence; in Italy t he percentage of smokers between 15 and 24 years is 24% [28].

For this reason primary prevention in schools setting is believed to be one of the most appropriate strategies to tackle substance use, also because schools offer a systematic and efficient way of reaching a large number of youngsters.

A recent Cochrane Review of school-based interventions to prevent drug use stated that only programs based on enhancing social skills have some chance of being effective [29]. The Cochrane report [30] affirms that it is necessary to support the programs based on the model of social influence with community interventions or with empowering social abilities to reinforce its effectiveness, because there is no strong evidence of the effectiveness of the interventions based only on the transmission of information.

Many studies [31] agree with World Health Organization (WHO) statement that it is important to develop Life Skills (LS) of young people to facilitate the development of the psychological skills that are required to deal with the demands and challenges of every day life.

For these reasons the OssFAD, in cooperation with other Italian Public Institutions, has produced the educational material for drug addictions prevention for the Italian primary, secondary and high schools [32].

The proposed methodology used to produce the educational material is innovative, because it starts from a perspective linked to the active participation of the beneficiaries. In fact in these last years new methodologies have been implemented which focused on the development of the life skills (decision making, critical thinking, problems solving, interpersonal relationship skills) and on the development of the competence of the beneficiaries [33].

The project was developed within several phases:

1. The collection of educational material on addiction prevention developed in Italy;
2. The identification of material that appraised the target of reference, the type of used language, the communication's effectiveness in comparison to the contents, the formative objectives;
3. The production of educational materials;
4. The set up of Italian schools database and the promotion of materials to schools;
5. The evaluation of the educational materials diffused administering a questionnaire to the teachers.

The Helpline Staff in this project has had a significant role in setting up the schools’ database, to promote educational material, to help the schools to develop the activities of prevention providing the learning material and indicate available sources to support the teaching activity. The Helpline Staff informed schools through a letter which explained the project and provided indications on the material and on the ways to obtain it.

A number of 3,521 primary, 2,231 secondary and 3,353 high schools were contacted, and out of those 4,098 schools (67.8% primary, 35% secondary and 31% high schools) all over Italy, answered and received the learning material. Answering a questionnaire on provided materials 80% of teachers agreed on usefulness of the materials, their pertinence and the easiness of proposed activities. For the teachers and the schools involved in this project, the Italian Antismoking Helpline has been an important institutional support easily available and free of charge.

### 2.5. Italian Antismoking Helpline and Anti-tobacco Media Campaigns

The major goal of the anti-tobacco media campaigns is to denormalise tobacco use in society and motivate current users to quit [34,35]. Media was the most important referral source for Helpline callers, followed by health care providers. The helplines, by their nature, are frequently used to provide information and to promote anti-tobacco media campaigns because, as above mentioned, they are services that can be more accessible to those who traditionally have not had access to health information [36].

When used in conjunction with mass media campaigns, a major challenge for helpline service providers is to maintain a high call answering rate, while providing at least minimal assistance to all callers [37].

The media campaigns involve making specific media spots to encourage tobacco users and others people to call the Helpline. The oragnizers of the media campaign considered it important to send a message, along with the anti-tobacco agenda, that assistance is available for those who want to quit but also for those who need information about tobacco-related issues.

To generate calls by different kinds of people the Italian Antismoking Helpline was involved in numerous media campaigns and other initiatives. Various mass media strategies were launched including newspaper articles, press releases, press interviews of investigators and counsellors, advertisements in local newspapers, feature articles.

**Figure 1.**
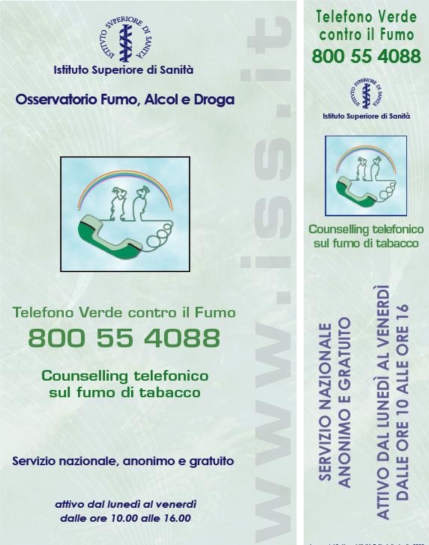
Leaflets and bookmarks of the Italian Antismoking Helpline.

Helpline posters, leaflets, and bookmarkers were designed and sent to various agencies to promote the service including schools, tertiary institutions, nongovernment organizations (NGO), public hospitals, healthcare professional organizations/associations, District Offices, and other Government Departments. Finally the OssFAD web site (www.iss.it/ofad) provides information about Helpline (Figure 1).

These campaigns were based on several concepts of social marketing; one such concept is the primary and secondary audiences [38]. In these campaigns, tobacco users who can be encouraged to call helplines are the primary audience, but there are important secondary audiences as well [38], such as tobacco users who may not call the helpline, but who will nonetheless make an attempt to quit as a result of the campaign. Friends and family members of tobacco users, local tobacco control advocates, health care providers, and policy makers make up another secondary audience for helpline. An effective marketing campaign will strive to obtain buy-in from this audience, because these individuals can help to encourage tobacco users to call.

Consequently, developing partnerships with organizations that represent members of these audiences is important because these groups can help to broadcast the quitline’s message to audiences that it might not otherwise reach. Moreover, as above mentioned, the secondary audiences can have a support for themselves from Helpline.

**Figure 2.**
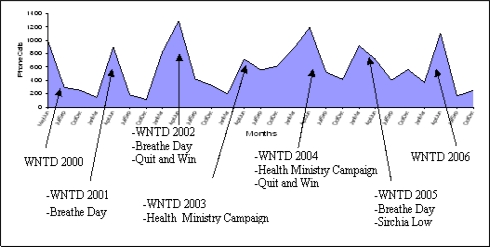
Monthly Trend of Phone Calls connected with main Italian Anti-tobacco Campaigns (May 2000 – December 2006).

It is hard to measure the effect of anti-tobacco media campaigns, of course, but our experience demonstrate that the number of calls received by the helpline and the kind of calls is very much influenced by media campaigns (Quit and Win, World No Tobacco Day, Health Ministry Campaigns, etc.). The Figure 2 shows the monthly trend of phone calls connected with main Italian Anti-Tobacco Campaigns.

In fact, the media campaigns and the helpline message can sometimes work together to produce a synergistic effect. An example is the coming into force in 2005 of Law 3 of 16th January 2003 regarding a smoking ban in all indoor public places (Sirchia Law). On this occasion the Italian Antismoking Helpline was involved to support the campaign to provide information about the law.

**Figure 3.**
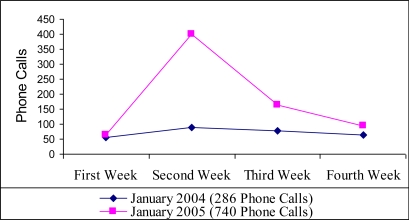
Comparison with number of calls received in January 2004 and in January 2005.

There were many mass media activities about the law and about the services to help people stop smoking; the Helpline’s telephone number was always mentioned in these activities. In this period there was a fourfold increase in the number of calls to the Italian Helpline, compared to the same week of the previous year (Figure 3).

The data shows that the variability in the number and kind of calls is connected with the attention given to the tobacco-related problems by health policies. Of course, the media campaign can affect not only those who actually call, but also those who don’t call, though it is hard to measure the effect of this case.

Ossip-Klein *et al*. [39] have shown, in a randomised study, which a majority of tobacco users who were informed of the existence of a helpline did not call for counselling services. However, the group that knew of the existence of a helpline was more likely to make an attempt to quit than the group that did not know about it. This suggests that it is beneficial to tag helpline telephone numbers to media happenings whenever it is appropriate.

## 3. Conclusions

The establishment of Italian Antismoking Helpline is a step but is not the solution to the smoking problem. Other strategies should be adopted by the Government, including raising the tobacco tax, strict enforcement on the prohibition of sale of tobacco to minors, enforcing strict school tobacco control policies, and banning all forms of tobacco advertisements and sponsorships (direct and indirect).

The Helpline is a complimentary strategy to an overall tobacco control policy and its effectiveness is related to the extent to which it provides accessible and acceptable quality services for smokers who wish to quit. Besides, it can provide correct and scientific information for other targets such as non smokers, health care professionals, teachers, social workers. Over the years the aid phone was used for a wide variety of issues and often the media health campaigns have made use of telephone counselling services for the many benefits arising out of the helpline.

As mentioned above, there are many reasons helplines have been so widely adopted. They are easy to promote and meet with broad acceptance by the public, because they eliminate barriers to access, such as lack of transportation and inability to pay for treatment, since the helplines have often a free telephone number.

For these and other reasons, Italian Antismoking Helpline has been assigned a central role in government sponsored tobacco cessation campaigns and serves as key component of comprehensive tobacco control programs [40]. Indeed the Antismoking Italian Helpline is set up in a Public Institution and the counsellors are not employed in the Helpline activity only, but also in others projects of the Italian Epidemiological Observatory on Tobacco, Alcohol and Drugs of abuse. Although it is not possible to perform a cost benefit analysis of the Italian Helpline because the data about quit rates are not available, it has to be recognized that this service is among a general antismoking policy which in the recent years succeeded in significantly decreasing the percentage of active smokers decreased in the last eight years from 28.9% to 22 % [6]. In 2007, more than 560,000 smokers quitted smoking leading to an increase in the percentage of ex-smokers from 17.5% to 18.4% in 2008 [28].

Although the Italian Helpline is best known for providing behavioural counselling to callers in developing and following a plan to quit smoking and to address smokers toward cessation services (SCS), it may also offer self help cessation literature, legal information and support to develop prevention activities in the school setting.

The mass media activities are the main channel of information about the existence of the Helpline; over the years there has been a steady increase of contacts by callers and a strong monthly variation in the number of calls received by the Italian Helpline. Antismoking Helplines are particularly useful if they are territory-related, and for Italian Antismoking Helpline the network represents not only a goal but a proper methodology to operate.

To promote health is fundamental for several socio-health agencies and the helplines may provide an initial access to information about health-related issues. In conclusion, a centralised helpline operation can be an accessible and effective service for a comprehensive tobacco control program.

## Figures and Tables

**Table 1.** Italian Antismoking Helpline aims and targets.

AIMS	TARGETS
✓ To give scientific information on effects produced by tobacco smoke, on possible anti-smoking therapies and on current legislation ✓ To carry out informative campaigns, formation and research activities ✓ To support and to promote relationship between different services ✓ To assess the Network of the Italian Cessation Services and to cooperate with them	✓ smokers: to support them in their effort to stop smoking and to help their families and/or friends in their support of the smoker ✓ no smokers: to suggest the strategies for protection from second- hand smoke ✓ health care professionals, social workers and teachers: to provide scientific and informative materials ✓ public and private institutions: to cooperate in carrying out studies and health protection campaigns

**Table 2.** Characteristics of Italian Antismoking Helpline callers.

*Callers’ Characteristics*	
*Gender*	%
Female	45.7
Male	54.3
*Groups of Callers*	%
Smokers	61.4
Non smokers	7.2
Ex-smokers	4.9
Family or friends	9.8
Other callers	17.9
*Geographic range of callers*	%
North	34.4
Center	23.2
South	17.9
Islands	9.2
Not found	13.9
*Main Areas of Interest*	%
Cessation Services	55.2
Psychological support	23.6
Anti-smoking therapies	10.1
Effects produced by tobacco smoke	2.4
Effects produced by second-hand smoke	2.6
Current legislation	6.6
Cooperation	5

## References

[1] Cornuz J, Zellweger JP, Burnard B (1994). Smoking cessation: importance for the patient and role of the practitioner. Schweiz. Med. Wochenschr.

[2] Franchini M, Caruso C, Perico A, Pacifici R, Monleon T, Garcia-Algar O, Rossi S, Pichini S (2008). Assessment of foetal exposure to cigarette smoke after recent implementations of smoke-free policy in Italy. Acta Paediatr.

[3] (2000). US Department of Health and Human Services. Reducing Tobacco Use: A Report of the Surgeon General. Department of Health and Human Services, Centers for Disease Control and Prevention.

[4] Pacifici R, Pichini S, Scafato E, Zuccaro P, Macchia T, Bartoli G, Di Pucchio A, Martucci L, Modigliani G, Mortali C, Pizzi E, Russo R (2001). Osservatorio su Fumo, Alcol e Droga. Not Ist Sup Sanità.

[5] WHO (2003). Draft WHO Framework Convention on Tobacco Control.

[6] (2008). Osservatorio Fumo, Alcol e Droga. Il fumo in Italia: Indagine DOXA 2008.

[7] Peto R, Lopez AD, Boreham J, Thun M (1994). Mortality From Smoking In Developed Countries 1950–2000.

[8] Peto R, Darby S, Deo H, Silcocks P, Whitley E, Doll R (2000). Smoking, smoking cessation, and lung cancer in the UK since 1950: combination of national statistics with two case-control studies. Brit. Med. J.

[9] Simonato L, Agudo A, Ahrens W, Benhamou E, Benhamou S, Boffetta P, Brennan P, Darby SC, Forastiere F, Fortes C, Gaborieau V, Gerken M, Gonzales CA, Jöckel KH, Kreuzer M, Merletti F, Nyberg F, Pershagen G, Pohlabeln H, Rösch F, Whitley E, Wichmann HE, Zambon P (2001). Lung cancer and sigarette smoking in Europe: an update of risk estimates and an assessment of inter-country heterogeneity. Int. J. Cancer.

[10] Strachan DP, Cook DG (1997). Health effects of passive smoking 1. Parental smoking and lower respiratory illness in infancy and early childhood. Thorax.

[11] He J, Vupputuri S, Allen K, Prerost MR, Hughes J, Whelton PK (1999). Passive smoking and the risk of coronary heart disease – A meta-analysis of epidemiologic studies. N. Engl. J. Med.

[12] Hackshaw AK, Law MR, Wald NJ (1997). The accumulated evidence on lung cancer and environmental tobacco smoke. BMJ.

[13] Windham GC, Eaton A, Hopkins B (1999). Evidence for association between environmental tobacco smoke exposure and birth weight: a metanalysis and new data. Paediatr Perinat. Epidemiol.

[14] Anderson HR, Cook DG (1997). Passive smoking and sudden infant death syndrome: review of the epidemiological evidence. Thorax.

[15] Fiore MC, Croyle RT, Curry SJ, Cutler CM, Davis RM, Gordon C, Healton C, Koh HK, Orleans CT, Richling D, Satcher D, Seffrin J, Williams C, Williams LN, Keller PA, Baker TB (2004). Preventing 3 million premature deaths and helping 5 millionsmokers quit: a national action plan for tobacco cessation. Am. J. Public Health.

[16] Hopkins DP, Briss PA, Ricard CJ, Husten CG, Carande-Kulis VG, Fielding JE, Alao MO, McKenna JW, Sharp DJ, Harris JR, Woollery TA, Harris KW (2001). Reviews of evidence regarding interventions to reduce tobacco use and exposure to environmental tobacco smoke. Am. J. Prev. Med.

[17] Lichtenstein E, Glasgow RE, Lando HA, Ossip-Klein DJ, Boles SM (1996). Telephone counseling for smoking cessation: rationales and meta-analytic review of evidence. Health Educ. Res.

[18] Stead LF, Lancaster T, Perera R (2004). Telephone counselling for smoking cessation (Cochrane Review). The Cochrane Library 2004.

[19] Zhu SH, Anderson CM, Johnson CE, Tedeschi G, Roeseler A (2000). A centralized telephone service for tobacco cessation: the California experience. Tob Control.

[20] Centers for Disease Control and Prevention Office on Smoking and Health.

[21] McAfee T, Sofian NS, Wilson J, Hindmarsh M (1998). The role of tobacco intervention in population-based health care: a case study. Am. J. Prev. Med.

[22] Zhu SH, Anderson CM, Jamner MS, Stokols D (2000). Bridging the clinical and public health approaches to smoking cessation: California Smokers’ Helpline. Promoting Human Wellness: New Frontiers for Research, Practice, and Polic.

[23] Zhu SH, Rosbrook B, Anderson C, Gilpin E, Sadler G, Pierce JP (1995). The demographics of help-seeking for smoking cessation in California and the role of the California Smokers’ Helpline. Tob. Control.

[24] Pacifici R, Di Pucchio A, Pizzi E, Martucci L, Mortali C, Zuccaro P (2001). L’attivita di counselling del Telefono Verde contro il Fumo dell’Istituto Superiore di Sanita. Boll Farmacodip e Alcoolis.

[25] Di Pucchio A, Pizzi E, Solimini R, Matrobattista L, Rossi S (2008). Structural and operational characteristics of Italian Smoking Cessation Services: a National investigation.

[26] Pizzi E Di Pucchio A Rossi S Carosi G Martucci L Mattioli D Mazzola M Mortali C Pacifici R Zuccaro P Guida ai servizi territoriali per la cessazione dal fumo di tabacco (Update 2007). Istituto Superiore di Sanità Roma, Italia 2008 (Strumenti di Riferimento 08/S1).

[27] Belleudi V Bargagli AM Davoli M Di Pucchio A Pacifici R Pizzi E Zuccaro P Peducci CAe gruppo di studio dei“Servizi territoriali per la cessazione dal fumo”. Interventi per la cessazione dell’abitudine al fumo in Italia: offerta ed efficacia nella pratica. Risultati di uno studio longitudinale multicentrico Epidemiol. Prev 2007 31 148 157 18677864

[28] Zuccaro P, Di Pucchio A, Pizzi E, Martucci L, Carosi G, Solimini R, Rossi S (2008). Il fumo in Italia. Respiro.

[29] Faggiano F, Vigna-Taglianti FD, Versino E, Zambon A, Borraccino A, Lemma P (2005). School-based prevention for illicit drugs' use. Cochrane Database Syst Rev.

[30] Thomas R, Perera R (2002). School-based programmes for preventing smoking. Cochrane Database Syst Rev.

[31] Botvin GJ, Griffin KW (2004). Life Skills Training: empirical findings and future directions. J. Prim. Prev.

[32] Zuccaro P, Caraffa G, Pizzi E, Di Pucchio A, Martucci L, Modigliani G, Rossi S, Mazzola M, Carosi G, Pichini S, Mattioli D, Pacifici R (2005). Venditori di Fumo. Conoscere i meccanismi che inducono al fumo di tabacco e le sue conseguenze. Percorso multimediale interattivo.

[33] NIDA Preventing Drug Use among Children and Adolescents. www.drugabuse.gov/pdf/prevention/inBrief.pdf.

[34] Stevens C (1998). Designing an effective counteradvertising campaign—California. Cancer.

[35] CDHHS/TCS (1998). A model for change: the California Experience in tobacco control.

[36] Bandura A (2001). Social Cognitive Theory of Mass Communication. Media Psychol.

[37] Wakefield M, Borland R (2000). Saved by the bell: the role of telephone helpline services in the context of mass-media anti-smoking campaigns. Tob. Control.

[38] Weinreich NK (1999). Hands-On Social Marketing: A Step-by-Step Guide.

[39] Ossip-Klein DJ, Giovino GA, Megahed N, Black PM, Emont SL, Stiggins J, Shulman E, Moore L (1991). Effects of a smokers’ hotline: results of a 10-county self-help trial. J. Consult. Clin. Psychol.

[40] Centers for Disease Control and Prevention (2004). Telephone quitlines: a resource for development, implementation, and evaluation.

